# Report on Typhoid Fever

**Published:** 1863-07

**Authors:** H. Noble

**Affiliations:** Heyworth, Ill.


### CHICAGO MEDICAL EXAMINER.

N. S. DAVIS, M.D., Editor.

VOL. IV. JULY, 1863. NO. 7.

### Original Contributions.

ARTICLE XV.

### REPORT ON TYPHOID FEVER.

By H. NOBLE, M.D., of Heyworth Ill.

Read to the Illinois State Medical Society.

Mr. President and Gentlemen:—The paper which I shall read to you is a "Report on Typhoid Fever," composed of remarks and observations on the nature and treatment of that disease.

This subject has, I know, been frequently and ably discussed: learned and minute essays from patient observers have from time to time been given, notwithstanding which, typhoid fever remains one of our most intractable diseases. I do not expect to elucidate in any great degree the obscurities of this disease, nor have I any special or new theory of its nature or cure. I only desire to give you in this report my own observations and conclusions, knowing well that from the sum of many observers' deductions the *truth* may, ultimately, be found.

My opinion is, that typhoid fever is a disease that is located, especially, in the mucous coat of the intestines; that in all cases of the disease there is irritation or inflammation of the mucous follicles, but not necessarily ulceration of Peyer's glands or any other tissue. Many cases of the disease, I think, show all the symptoms of typhoid fever, and are fully matured without ulceration.

When the disease is uncomplicated, the patient, generally, complains for several days of slight indisposition only, the appetite sometimes not materially impaired, yet there is an evident want of nutrition. Before there is any abdominal tenderness, the tongue indicates mucous irritation, the tips and edges being red; and, as the disease progresses, the redness increases and becomes deeper, the surface becomes glossy and smooth, often with the papilla enlarged.

Unless modified by treatment, the bowels become tumefied and tender, on pressure, diarrhœa usually attending, in some cases the action of the bowels remain natural. The pulse is, generally, accelerated, sometimes to 150 to the minute, this is not invariably the case.

I have seen five or six cases of typhoid fever, in which, the pulse did not, for the first four weeks, exceed 50 beats to the minute, at any time that I observed it. One of these cases terminated fatally, after five weeks' duration, the pulse, during the last eight or ten days, reaching 120. As the other cases got well, the pulse increased in frequency until it reached the natural standard, but never exceeded it during the continuance of the disease.

In the fatal cases which I have seen, death resulted from the following causes, viz.:—Perforation of the intestine, prostration or exhaustion of the vital forces, and from the complication of other diseases. Perforation of the bowel, though not very common, is *very fatal*, no case that I have seen surviving more than thirty or forty hours after the occurrence took place. When perforation of the bowel occurs, it is attended with symptoms of peritonitis; the agonized expression of countenance being always present; pulse rapid and feeble; excessive restlessness; cold sweat; and rapid prostration.

The collapse which follows perforation of the bowel, in typhoid fever, resembles, in every respect, so far as I can judge, the collapse of Asiatic cholera,—the want of capillary circulation is shown by the blue color of the skin being alike in both cases. The patient, after perforation, is sensible of pain for a short time only, say from two to six hours, after which, the nerves loose their normal action and do not respond to the stimulus which usually excites sensation. Those cases which died of prostration, had been sick from six to twelve weeks, and were attended with a general failure of the vital forces. We sometimes see cases begin, and progress in a mild form, and without complication, yet the patient becomes emaciated and feeble, nutrition being much impaired, if not entirely suspended, and the patient dies without the supervention of any active disease; but by far the greatest number of deaths that have occurred under my observation, have resulted from complication with other diseases.

The most fatal of these complications is the occurrence of inflammation in the cerebral cavity. This may occur at any period of the disease, though the cases that I have witnessed, with few exceptions, took place early, say from the third to the sixth day. Those cases commenced and progressed for two or three days without apparent danger or violence, the patient thinking himself not sick enough to need a physician, until a partial coma alarms the friends enough to call a physician. The coma increases until it is difficult to arouse the patient, who is continually reaching for and catching at imaginary objects, pulling the bed-clothes and muttering to himself disconnected words and sentences. When typhoid fever becomes complicated with congestion or inflammation of the brain, the latter becomes the leading or principal disease, its symptoms being far more prominent and alarming than the fever which preceded. Of this class of cases, I have only seen a few recover; but, when convalescence was established, it was at an earlier period than would have been expected had the fever progressed without complication. That is, when the cerebral symptoms are relieved, the fever does not recur. I shall have a word to say on this subject under the head of hemorrhage.

Pneumonia is a more common though less fatal complication, a large proportion of typhoid cases recovering after its occurrence. It takes place in any stage of typhoid fever, though I

believe it is most common in the early or forming stage. Although the occurrence of pneumonia adds much to the peril of the typhoid patient, a large proportion of cases recover after its attack. Another complication or, perhaps, more properly, a consequence of typhoid fever is hemorrhage from the bowels. This is not so frequently met with as pneumonia, is much more dangerous, generally prostrating the patient in a few hours past all possibility of recovery. I have never seen hemorrhage from the bowels occur in typhoid fever until the latter disease was fully developed; and although large quantities of blood are sometimes discharged, I do not think it necessarily the result of organic lesion, relaxation of the mucous membrane being sufficient to allow any quantity of blood to escape, which we ever see evacuated by the bowels.

As mentioned under the head of cerebral complications, the primary discharge sometimes is relieved by the evacuation of blood from the bowels. It is, perhaps, easier to understand how the effusion of blood from the mucous membrane should relieve the system of disease supposed to be located in that membrane, than it is to see how inflammation or disease, in an organ remote from the primary seat of disease, should relieve that primary disease; but when we consider that the whole system is nurtured by nerves which spring from one centre, the mystery will as once be solved. An impression made on the nervous centre, either by disease or remedies, affects the whole nervous system, and may render the whole, or any part of it, unsusceptible to a local influence. Thus, typhoid fever may be rendered powerless by the influence of another disease acting through the medium of the nerves. I have no doubt that typhoid fever is frequently eradicated from the system in that way; but I do not consider it a desirable or safe way, as I have only seen one patient recover after blood in large quantity had been evacuated from the bowels.

The above-enumerated are some of the principal diseases with which typhoid fever may be complicated, but by no means all of them. I have only mentioned the most important, as regards the frequency of their occurrence, and their danger when present. Any person will readily perceive that typhoid fever has, by its first morbid effect on the system, (namely, arresting, partially, nutrition,) not only rendered the system less able to resist the attack of a new disease, but it had also, in a measure, destroyed the restorative power, by reducing, through the want of nutrition, the vital forces. Under such circumstances, what is generally considered quite light and unimportant disease, assumes, to the experienced observer, a very grave importance; and he is prepared to see the system yield to ailments which, in a person in general good health, scarcely require medical interference.

In forming our prognosis of any case of local disease, we take into consideration the importance of the organ or tissue implicated, from which, with the amount of disease present or expected, we form our opinion of the result.

Now, if the view I have taken of typhoid fever be correct, the tissue implicated is a portion of the alimentary canal, which forms an important part of the apparatus that nourishes and sustains the whole system. There are then few diseases which are located in more important tissues than typhoid fever itself; and, when we consider the frequency of its complications and the inability of the system, when under its influence, to resist disease, we must say, that typhoid fever stands at or very near the head of formidable diseases in this country. I believe the disease is, through the country, generally, on the increase, some localities, however, being more subject to it than others. The towns of Newcastle, and Waynesville, and adjacent country were first visited with typhoid fever in 1846 and '7, at which time it was fatal in a large majority of the cases which occurred. Since then, it has been met with in all parts of the county, but it is more prevalent in some parts than in others. For instance, there has been for several years past more typhoid fever on Salt Creek, a stream which rises in or near the N.E. corner of McLean Co., than there has been in any other section of country with which I am acquainted. I do not know any local cause for the greater prevalence of the disease in that locality. I am not acquainted with the geological formation of the country well enough, to say there is no difference between that and more healthy localities; but, if there be a difference, it is not apparent to a superficial observer. I believe autumnal diseases are no more frequent in that region than in others.

That the geological formation of a country modifies the diseases of the same, I have no doubt. The army of the frontier has suffered this spring (1863,) from a disease which the surgeons call typhoid pneumonia, the fatality of which was truly appalling. The symptoms were, rigors, congestion of the lungs and brain, coma, and *death*,—many cases terminating fatally in less than forty hours. In the cases described, congestion was the leading symptom; but, in all cases where the disease was checked or arrested, the typhoid character was apparent; and, although the typhoid condition might be supposed to be the result of the congestion, the surgeons, generally, thought that the latter was the congestive stage of the typhoid disease.

Now, the geological formation there (the Ozark mountain region,) is entirely different from *our Illinois*. The soil is very loose and sandy, mixed with small irregular shaped limestone, giving it great facility to absorb water, and the same conditions being favorable for great evaporation. I should look for autumnal diseases to be prevalent on such a formation; and our army has found that the spring season is not exempt from serious disease. While autumnal diseases, such as diarrhœa, dysentery, intermittent and bilious fever have been prevalent in the army of the frontier, uncomplicated typhoid fever has not prevailed to any great extent, there being, perhaps, fewer cases than might have been expected in the same population at home.

In the treatment of this disease, I differ with many of my friends in the profession. I know that many physicians profess to *cure* typhoid fever; some of them say, that from four to six days is as long as it generally runs with them, unless it had progressed to something like maturity before they had it under treatment; but I also know that that degree of success is not universally allowed by the profession. The typhoid fever that has come under my observation has always been an obstinate disease, running from three to twelve weeks to establish convalescence, no case yielding, according to my recollection, in less than twenty-one days. This is very different from the opinions expressed by many of our friends who have written on the subject, who state, that, by a particular treatment, they, generally, cut the disease short,—that is, cure it in a few days.

The plan of treatment which I have followed for several years past has been especially directed, first, to the correction of the supposed lesion of the intestines; and, next, to sustain the strength and vitality of the patient. After evacuating the bowels with a mercurial preparation, with which I frequently combine from one to three grains of quinine, I give nitric acid diluted to about the strength of good vinegar, in teaspoonful doses, repeated in four or six hours, according to the urgency of the case. Should the tongue become red or shining, I give turpentine in from twenty to sixty drop doses from two to four times a day, until the tongue resumes its natural color or until the unnatural redness disappears, after which, I resume the use of the nitric acid, and continue it, generally, until convalescence is established, unless it becomes necessary to suspend it to use other remedies. I have observed, that, when the redness of the tongue has been corrected by the use of turpentine, the exhibition of the acid prevented its recurrence, in some cases, through the whole disease, and, in all, its effects seemed to be beneficial.

I do not think that nitric acid possesses any specific power to control the intestinal disease, but benefits the patient by its tonic and invigorating action, thereby enabling the system more effectually to resist the influence of disease and, at the same time, promote the recuperative process. At any time, during the progress of the disease, that the secretions become deranged or deficient, I give calomel sufficient to restore them as near as possible, always discontinuing its use as soon as that particular object is attained. During the whole course of the disease, I consider it necessary to watch carefully the appearance of any new disease; and, if any such occurs, to relieve by judicious means, as soon as possible, the patient from the unpleasant complication.

To sustain as much as possible the strength of the patient, the diet should be nourishing, easily digested, and not stimulating. It should also be made up of a variety of dishes,—never allowing the patient to make a meal off one course. It should be taken, as near as possible, at the accustomed hour for meals, as it is thought that the digestion is more active at the regular periods than at other times.

Under this mode of treatment, this disease, although a formidable and dangerous one, is not more fatal than many other diseases which have less reputation for obstinate fatality. I cannot give the per cent of the fatal out of the whole number of cases, but I do not think it will exceed the fatality of pneumonia or dysentery.

